# A comparative evaluation of publicly available large language models in the assessment of CTG traces according to the FIGO criteria

**DOI:** 10.1007/s00404-025-08145-w

**Published:** 2025-08-21

**Authors:** Iason Psilopatis, Cécile Monod, Valeria Filippi, Rebecca Tschudin, Olaf Lapaire, Julius Emons, Beatrice Mosimann, Tibor A. Zwimpfer

**Affiliations:** 1 Department of Gynecology and Obstetrics, University Hospital Basel, University of Basel, Basel, Switzerland; 2 Department of Gynecology and Obstetrics, Universitätsklinikum Erlangen, Comprehensive Cancer Center Erlangen-EMN (CCC ER-EMN), Friedrich-Alexander-Universität Erlangen-Nürnberg (FAU), Erlangen, Germany; 3 Department of Biomedicine, University Hospital and University of Basel, Basel, Switzerland

**Keywords:** Artificial intelligence, Large language model, CTG, Obstetrics, Fetal monitoring

## Abstract

**Background:**

Cardiotocography (CTG) remains a cornerstone in fetal monitoring, but its interpretation is subject to considerable inter- and intra-observer variability. Artificial intelligence (AI) tools, particularly large language models (LLMs), offer potential to improve diagnostic consistency and reduce clinician workload.

**Objectives:**

This study aims to evaluate and compare the accuracy of various LLMs in CTG interpretation based on Federation of Gynecology and Obstetrics (FIGO 2015) criteria.

**Study design:**

An analysis of sixty CTG traces previously classified by clinicians at the University Hospital Basel according to FIGO guidelines was conducted. In a two-run protocol, 30 normal CTG traces were initially presented as screenshots to Chat-GPT-4.0, Google Gemini, Bing Copilot, and DeepSeek. Subsequently, the LLMs that demonstrated adequate interpretation of normal CTGs were tasked to classify another 30 suspicious or pathological CTG traces. Each LLM was asked to classify each CTG trace as normal or abnormal.

**Results:**

DeepSeek was unable to interpret CTGs and was excluded. Google Gemini showed poor performance (6.7%) on normal CTGs. Chat-GPT-4.0 partially succeeded in correctly classifying the provided CTG traces as normal (46.7%) or abnormal (50%). Bing Copilot accurately interpreted normal CTGs (96.6%) but failed on abnormal ones (0%).

**Conclusions:**

LLMs show major limitations in the interpretation of CTG traces according to the FIGO criteria.

**Supplementary Information:**

The online version contains supplementary material available at 10.1007/s00404-025-08145-w.

## What does this study add to the clinical work?

**Table:** 

LLMs show major limitations in the interpretation of CTG traces according to the FIGO criteria. Their integration into routine CTG analysis may not yet be supported.	

## Introduction

Cardiotocography (CTG) remains a cornerstone of modern intrapartum fetal surveillance, enabling a continuous, non-invasive monitoring of fetal heart rate (FHR) and uterine contractions to detect fetal compromise [1]. By identifying early signs of hypoxia, CTG aims to guide timely interventions that reduce the risk of adverse perinatal outcomes such as hypoxic-ischemic encephalopathy and stillbirth [2]. To reduce the inherent subjectivity in CTG interpretation, the international Federation of Gynecology and Obstetrics (FIGO 2015) developed standardized classification criteria, categorizing CTG traces as normal, suspicious, or pathological based on FHR baseline, variability, decelerations, and uterine contractions [3, 4]. While these guidelines improve consistency, interpretation remains highly dependent on clinician experience, and inter-observer variability persists, particularly in ambiguous or borderline cases [5].

Artificial intelligence (AI) offers promising solutions for enhancing diagnostic consistency and decision-making across medical disciplines [6]. Among the diverse AI modalities, large language models (LLMs) have shown strong capabilities in processing complex textual and contextual information [7]. These models, trained on vast datasets of medical literature, clinical guidelines, and patient records, possess the potential to augment clinical decision-making by providing evidence-based insights and automating routine tasks [8]. In obstetrics and gynecology, LLMs like Chat-GPT have demonstrated potential in accurately interpreting guidelines, formulating appropriate management decisions in simulated scenarios and generating structured recommendations comparable to those of experienced clinicians [9–12]. By leveraging their ability to extract and synthesize information from diverse sources, LLMs could potentially improve the accuracy and consistency of patient data interpretation, reducing the cognitive workload, and standardizing interpretations, particularly in time-sensitive, high-stakes settings [13]. The application of LLMs to medical data analysis is still in its infancy, but the initial results demonstrate a potential to reshape the clinical workflow [14].

This study aims to investigate the potential of four different LLMs to interpret CTG traces. We plan to evaluate their ability to classify traces according to FIGO guidelines and compare their performance to that of human experts.

## Materials and methods

### Study design and setting

This was an observational study designed to assess the performance of LLMs in interpreting CTG traces in comparison to expert clinical evaluations. The study was conducted at the Department of Gynecology and Obstetrics, University Hospital Basel, Switzerland. All CTG recordings were retrieved from the institution’s electronic medical records system. Ethical approval for this study was granted by the Ethical Committee of Nordwest und Zentralschweiz, Switzerland (Approval Number: 2025-00536).

### Data sources and variables

All sixty CTG recordings were pre-classified by two obstetricians with over 10 years of clinical experience using the FIGO 2015 guidelines (normal, suspicious, pathological) [3]. In cases of initial disagreement, a consensus was reached through discussion to establish the final reference classification. These expert-labeled classifications served as the reference standard against which LLM output were compared.

### Participants

A total of sixty CTG traces recorded during routine clinical care in April 2025 were included. These sixty traces were divided into two sets of thirty for the two study runs.

Eligibility criteria were:

-Singleton pregnancies monitored between 37 + 0 and 42 + 0 weeks of gestation

-Complete CTG traces of at least 30 min duration

-Availability of clinician-assigned FIGO classification at the time of delivery

Exclusion criteria included:

-Incomplete or corrupted CTG data

-Missing or inconsistent FIGO classification

### Intervention and exposure

Four distinct LLMs were assessed in this study:

-Chat-GPT-4.0 (OpenAI)

-Gemini 2.0 (Google)

-Bing Copilot (Microsoft)

-DeepSeek

Each model was provided with identical prompts and CTG information and asked to classify traces according to FIGO criteria. The prompt explicitly defined the color coding of the traces (“Red is fetal heart rate, pink is maternal heart rate and black uterine contractions”) to ensure the LLMs correctly identified each component, given their primary training on textual data and the visual nature of CTGs presented as images. No other information or clinical context (e.g., patient demographics, medical history) was provided to the LLMs. Similarly, the expert clinicians who provided the reference classifications interpreted the CTG traces without access to additional clinical data beyond the visual CTG itself.

### Study phases

The experimental procedure was conducted in two distinct runs:

Run 1: Thirty CTG traces, previously classified as FIGO normal by human experts, were presented as screenshots to each of the four LLMs (Chat-GPT-4.0, Google Gemini, Bing Copilot, and DeepSeek). The LLMs were instructed to analyze each CTG trace and to provide a classification based on the FIGO guidelines. It is important to note that the LLMs were not informed that these CTGs had been pre-classified as “normal” by the experts; their task was to perform an unsupervised classification based solely on the provided image and prompt.

Run 2: Based on the results of the first run, only the LLMs that demonstrated (partially) adequate interpretation of the FIGO normal CTGs were selected for the second run. In this run, thirty CTG traces, previously classified as FIGO suspicious or pathological, were presented to the LLMs Chat-GPT and Bing Copilot. These LLMs were again instructed to analyze and classify the CTG traces according to the FIGO guidelines.

### Data analysis

The primary outcome was classification accuracy compared to the reference standard classified by two senior obstetricians at the University Hospital Basel. Secondary outcomes included sensitivity and specificity for detecting suspicious/pathological CTGs. Descriptive statistics were used to assess performance across models. No formal hypothesis testing was performed due to the exploratory nature of the study.

### Bias and missing data

Selection bias was minimized by including all eligible CTG traces from a defined time period. There were no missing data in the final dataset, and all included traces were interpretable by human experts; therefore, no percentage of missing signal was calculated.

### Study size

No formal sample size calculation was conducted, as this was a pilot study intended to evaluate feasibility and comparative performance across models.

## Results

### First run: evaluation of FIGO normal CTGs

The initial phase evaluated the performance of the four LLMs Chat-GPT-4.0, Google Gemini, Bing Copilot, and DeepSeek in classifying 30 CTG traces previously assessed by the obstetricians as FIGO normal.

DeepSeek was unable to process or interpret any CTG trace due to the visual nature of CTGs presented as images and was therefore excluded from further analyses.

Google Gemini misclassified 28/30 (93.3%) CTG traces as non-reassuring, mostly due to variable decelerations, despite normal baseline and variability (Table 1).

**Table 1:** Google Gemini’s potential to evaluate FIGO normal CTGs

CTG No.	Baseline FHR	Variability	Accelerations	Decelerations	Overall interpretation
1	Normal	Normal	Present	Variable	Unclear
2	Normal	Normal	Present	Variable	Non-reassuring
3	Normal	Normal	Present	Variable	Non-reassuring
4	Normal	Normal	Present	Uncomplicated variable	Reassuring
5	Normal	Normal	Present	Variable	Non-reassuring
6	Normal	Normal	Present	Variable	Non-reassuring
7	Normal	Normal	Present	Variable	Non-reassuring
8	Normal	Normal	Present	Variable	Non-reassuring
9	Normal	Normal	Present	Variable	Non-reassuring
10	Normal	Normal	Present	Variable	Non-reassuring
11	Normal	Normal	Present	Variable	Non-reassuring
12	Normal	Normal	Present	Variable	Non-reassuring
13	Normal	Normal	Present	Variable	Non-reassuring
14	Normal	Normal	Present	Variable	Non-reassuring
15	Normal	Normal	Present	Variable	Non-reassuring
16	Normal	Normal	Present	Variable	Non-reassuring
17	Normal	Normal	Present	Variable	Non-reassuring
18	Normal	Normal	Present	Variable	Non-reassuring
19	Normal	Normal	Present	Variable	Non-reassuring
20	Normal	Normal	Present	Variable	Non-reassuring
21	Normal	Normal	Present	Variable	Non-reassuring
22	Normal	Normal	Present	Variable	Non-reassuring
23	Normal	Transient tachycardia	Present	Variable	Non-reassuring
24	Normal	Normal	Present	Variable	Non-reassuring
25	Normal	Normal	Present	Variable	Non-reassuring
26	Normal	Normal	Present	Variable	Non-reassuring
27	Normal	Normal	Present	Variable	Non-reassuring
28	Normal	Normal	Present	Variable	Non-reassuring
29	Normal	Normal	Present	Variable	Non-reassuring
30	Normal	Normal	Present	Variable	Non-reassuring

Chat-GPT-4.0 also misclassified 16/30 (53.3%) CTG traces as suspicious, largely because of (recurrent) variable decelerations (Table 2).

**Table 2:** Chat-GPT’s potential to evaluate FIGO normal CTGs

CTG No.	Baseline FHR	Variability	Accelerations	Decelerations	Overall interpretation
1	Normal	Normal	Present	Occasional, non-pathological	Reassuring
2	Normal	Normal	Occasional	Minor variable, no pathological	Reassuring
3	Normal	Brief early reduction	Present	Minor variable, no pathological	Reassuring
4	Normal	Borderline	Absent	Minor, non-pathological	Suspicious
5	Normal	Normal	Present	One variable	Suspicious
6	Normal	Normal	Present	None pathological	Normal
7	Normal	Normal	Present	None pathological	Normal
8	Normal	Normal	Present	None pathological	Normal
9	Normal	Normal	Present	Isolated variable, non-pathological	Normal
10	Normal	Normal	Present	Isolated variable, non-pathological	Normal
11	Normal	Normal	Present	Isolated variable	Normal
12	Normal	Normal	Present	Multiple variable, non-pathological	Suspicious
13	Normal	Normal	Present	Recurrent variable	Suspicious
14	Normal	Normal	Present	Recurrent variable	Suspicious
15	Normal	Normal	Present	Recurrent variable	Suspicious
16	Normal	Normal	Present	None pathological	Normal
17	Normal	Normal	Present	None	Normal
18	Normal	Normal	Present	Recurrent variable	Suspicious
19	Normal	Normal	Present	None	Normal
20	Normal	Normal	Present	None pathological	Normal
21	Normal	Normal	Present	Recurrent variable	Suspicious
22	Normal	Normal	Present	Recurrent variable	Suspicious
23	Normal	Normal	Present	Recurrent variable	Suspicious
24	Normal	Normal	Present	Recurrent variable	Suspicious
25	Normal	Normal	Present	None pathological	Normal
26	Normal	Normal	Present	Recurrent variable	Suspicious
27	Normal	Normal	Present	Recurrent variable	Suspicious
28	Normal	Normal	Present	Recurrent variable	Suspicious
29	Normal	Normal	Present	Recurrent variable	Suspicious
30	Normal	Normal	Present	Recurrent variable	Suspicious

In contrast, Bing Copilot correctly identified 29/30 (96.6%) CTG traces as normal, demonstrating strong alignment with obstetrician-assigned classifications and accurate application of FIGO guidelines (Table 3).

**Table 3:** Bing Copilot’s potential to evaluate FIGO normal CTGs

CTG No.	Baseline FHR	Variability	Accelerations	Decelerations	Overall interpretation
1	Normal	Normal	Present	Uncomplicated Variable	Reassuring
2	Normal	Normal	Present	None significant	Reassuring
3	Normal	Normal	Present	None significant	Reassuring
4	Normal	Normal	Present	Uncomplicated Variable	Reassuring
5	Normal	Normal	Present	Variable	Unclear
6	Normal	Normal	Present	None significant	Reassuring
7	Normal	Normal	Present	None significant	Reassuring
8	Normal	Normal	Present	Uncomplicated Variable	Reassuring
9	Normal	Normal	Present	None significant	Reassuring
10	Normal	Normal	Present	None significant	Reassuring
11	Normal	Normal	Present	None significant	Reassuring
12	Normal	Normal	Present	None significant	Reassuring
13	Normal	Normal	Present	None significant	Reassuring
14	Normal	Normal	Present	None significant	Reassuring
15	Normal	Normal	Present	None significant	Reassuring
16	Normal	Normal	Present	None significant	Reassuring
17	Normal	Normal	Present	None significant	Reassuring
18	Normal	Normal	Present	None significant	Reassuring
19	Normal	Normal	Present	None significant	Reassuring
20	Normal	Normal	Present	None significant	Reassuring
21	Normal	Normal	Present	None significant	Reassuring
22	Normal	Normal	Present	None significant	Reassuring
23	Normal	Normal	Present	None significant	Reassuring
24	Normal	Normal	Present	None significant	Reassuring
25	Normal	Normal	Present	None significant	Reassuring
26	Normal	Normal	Present	None significant	Reassuring
27	Normal	Normal	Present	None significant	Reassuring
28	Normal	Normal	Present	None significant	Reassuring
29	Normal	Normal	Present	None significant	Reassuring
30	Normal	Normal	Present	None significant	Reassuring

**Fig. 1:**
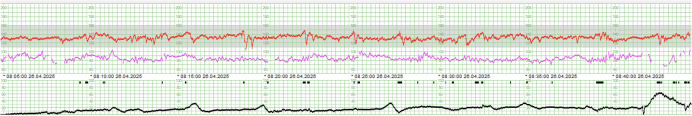
LLM’s potential to evaluate FIGO normal CTGs

Figure 1 depicts the responses of the different LLMs to one of the investigated CTG of the study cohort with normal traces.

Supplementary File 1 includes all thirty studied normal CTG traces.

### Second run: evaluation of FIGO suspicious/pathological CTGs

Building upon the insights gained from the first run, the second phase of the study focused on evaluating the performance of Chat-GPT and Bing Copilot—the two LLMs that (partially) successfully interpreted the normal CTGs—in classifying a new set of 30 CTG traces that had been classified as FIGO suspicious or pathological by clinical experts.

Bing Copilot failed to classify any of the 30 suspicious/pathological CTG traces correctly, mislabeling all as reassuring (24/30) or unclear (6/30) (Table 4).

**Table 4:** Bing Copilot’s potential to evaluate FIGO abnormal CTGs

CTG No.	Baseline FHR	Variability	Accelerations	Decelerations	Overall interpretation
1	Normal	Moderate	Present	None significant	Reassuring
2	Normal	Moderate	Present	None significant	Reassuring
3	Normal	Moderate	Present	None significant	Reassuring
4	Normal	Moderate	Present	None significant	Reassuring
5	Normal	Moderate	Present	None	Reassuring
6	Normal	Moderate	Present	None significant	Reassuring
7	Normal	Moderate	Present	Present	Unclear
8	Normal	Moderate	Present	None significant	Reassuring
9	Normal	Moderate	Present	None significant	Reassuring
10	Normal	Moderate	Present	Present	Unclear
11	Normal	Moderate	Present	Variable	Reassuring
12	Normal	Moderate	Present	Single variable	Reassuring
13	Normal	Moderate	Present	Late	Unclear
14	Normal	Moderate	Present	Variable	Unclear
15	Normal	Moderate	Present	Early and variable	Unclear
16	Normal	Moderate	Present	Variable	Reassuring
17	Normal	Moderate	Present	Variable	Reassuring
18	Normal	Moderate	Present	Variable	Unclear
19	Normal	Moderate	Present	None	Reassuring
20	Normal	Moderate	Present	Variable	Reassuring
21	Normal	Moderate	Present	Variable	Reassuring
22	Normal	Moderate	Present	None significant	Reassuring
23	Normal	Moderate	Present	Variable	Reassuring
24	Normal	Moderate	Present	Variable	Reassuring
25	Normal	Moderate	Present	None	Reassuring
26	Normal	Moderate	Present	Variable	Reassuring
27	Normal	Moderate	Present	None	Reassuring
28	Normal	Moderate	Present	None significant	Reassuring
29	Normal	Moderate	Present	Variable	Reassuring
30	Normal	Moderate	Present	Variable	Reassuring

Chat-GPT correctly classified only half of the provided CTG traces (15/30) as suspicious or pathological (Table 5).

**Table 5:** Chat-GPT’s potential to evaluate FIGO abnormal CTGs

CTG No.	Baseline FHR	Variability	Accelerations	Decelerations	Overall interpretation
1	Normal	Normal	Present	Variable, acceptable	Normal
2	Normal	Normal	Present	Variable, occasional deep ones	Suspicious
3	Normal	Normal	Present	Early decelerations	Normal
4	Normal	Normal	Present	Variable, acceptable	Normal
5	Normal	Normal	Present	Variable, acceptable	Normal
6	Normal	Normal	Present	Variable, acceptable	Normal
7	Normal	Reduced (intermittent)	Present	Variable, some deep and prolonged, concerning	Pathological
8	Normal when visible, but unstable	Reduced after decelerations	Absent	Frequent, prolonged, late and atypical variable	Pathological
9	Normal	Normal	Present	Variable, acceptable	Normal
10	Unstable, concerning	Reduced	Absent	Recurrent deep, prolonged, late/atypical	Pathological
11	Normal	Absent	Absent	None significant	Pathological
12	Normal	Normal	Absent	Single prolonged (> 3 min), deep, recovered	Pathological
13	Unclear, intermittent when visible	Reduced (brief)	Absent	Recurrent, deep, atypical variable and prolonged	Pathological
14	Normal	Normal	Present	Variable, frequent but recovering	Suspicious
15	Normal	Normal to intermittently reduced	Present	Recurrent atypical variable and prolonged	Pathological
16	Normal	Normal to reduced	Present	Recurrent atypical variable, some prolonged	Pathological
17	Normal	Normal	Present	Variable, acceptable	Suspicious
18	Normal	Normal	Present	None significant	Normal
19	Normal	Normal	Present	Variable, acceptable	Normal
20	Normal	Normal	Present	Variable, acceptable	Normal
21	Normal	Reduced after decelerations	Present	Recurrent, deep, atypical variable	Pathological
22	Normal	Normal	Present	Recurrent variable, some atypical	Suspicious
23	Normal	Normal	Present	Variable, shallow, quick recovery	Normal
24	Normal	Normal	Present	Variable, acceptable	Normal
25	Normal	Normal	Present	Variable, shallow to moderate	Normal
26	Normal	Normal	Present	Variable, shallow to moderate	Normal
27	Normal	Normal	Present	Variable, shallow-moderate	Normal
28	Unstable	Reduced	Absent	Recurrent deep atypical, some prolonged	Pathological
29	Normal	Normal	Present	Variable, acceptable	Normal
30	Normal but interrupted	Reduced intermittently	Few	Recurrent deep atypical variable and prolonged	Pathological

**Fig. 2:**
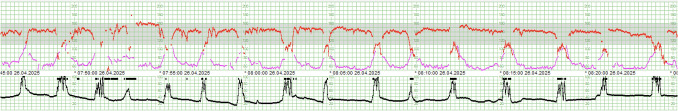
LLM’s potential to evaluate FIGO abnormal CTGs

Figure 2 depicts the responses of the LLMs to one of the investigated CTG of the study cohort with abnormal traces.

Supplementary File 2 includes all thirty studied abnormal CTG traces.

## Discussion

### Principal findings

This study provided an initial assessment of the capabilities of contemporary LLMs in classifying CTG traces according to established FIGO criteria, using expert clinician classifications as the reference standard. DeepSeek was unable to interpret the CTG data entirely. While Google Gemini demonstrated poor performance on normal CTGs, Bing Copilot showed high accuracy in classifying normal CTGs but failed entirely on abnormal traces. Chat-GPT-4.0 exhibited partial success in classifying both normal and abnormal CTGs, but with a substantial error rate. Overall, the findings indicate that current general-purpose LLMs face substantial limitations in accurately interpreting CTG traces based on FIGO criteria.

## Results

Compared to prior studies, which primarily focused on classical machine learning algorithms for CTG analysis [15, 16], this study uniquely explores the potential of LLMs, a class of AI tools with natural language processing capabilities that allow not only pattern classification but also clinical reasoning and explanation. Earlier work has demonstrated the promising results of machine learning algorithms in automating CTG analysis and improving diagnostic accuracy in the context of AI-driven computerized CTG analysis [17, 18]. However, LLMs offer the added advantage of interpretability through text-based justifications, enhancing transparency and facilitating validation. Notably, this study contrasts with recent findings by Gumilar et al. who concluded that Chat-GPT-4.0 outperformed Gemini Advanced and Copilot in CTG interpretation [19]. Our analysis did not replicate those results in a larger and clinically validated dataset. The discrepancy may stem from differences in methodology, particularly the type and number of CTG traces used for evaluation, and the specific prompts provided. Gumilar et al.’s study might have utilized a different selection of CTG traces or a prompt strategy that better aligned with the LLMs’ internal knowledge representations, leading to varied performance outcomes. Our study focused on providing raw visual screenshots and a standardized FIGO prompt, which might have exposed the inherent limitations of current general-purpose LLMs in visually driven medical interpretation.

### Clinical implications

The observed limitations of the LLMs in accurately interpreting CTG traces according to FIGO criteria likely stem from several factors. First, the inherent complexity and visual nature of CTG interpretation, which relies on recognizing patterns and trends in continuous data rather than discrete textual information, may pose a significant challenge for models primarily trained on language. While the prompts provided textual descriptions of the expected classifications, the LLMs lacked direct access to the visual representations of the CTG traces themselves. This absence of visual input likely hindered their ability to identify subtle but clinically significant features, such as baseline variability, the morphology of decelerations, and the context of uterine contractions in relation to fetal heart rate changes. Second, the FIGO guidelines, while standardized, still involve a degree of clinical judgment and contextual awareness that may be difficult to fully codify into prompts for an AI. The distinction between normal physiological variations and early signs of fetal distress can be nuanced and requires integrating multiple parameters over time, a capability that current LLMs may not possess to the same extent as experienced clinicians. Finally, the training data for these general-purpose LLMs may not include a sufficient quantity or quality of examples specifically linking visual CTG patterns to FIGO classifications, leading to a lack of specialized knowledge in this domain.

### Research implications

Future research should focus on addressing the identified limitations to enhance the potential of AI in CTG interpretation. One key direction involves exploring multimodal approaches that enable LLMs or specialized AI models to directly process visual representations of CTG traces, potentially through integration with computer vision techniques. Furthermore, the development of meticulously curated and annotated datasets of CTG traces, linked to FIGO classifications and clinical outcomes, is crucial for training more accurate and reliable AI models. Investigating the impact of different prompting strategies and the incorporation of more detailed clinical context into the AI analysis could also improve performance. For instance, testing whether LLM performance improves after receiving corrective feedback on misclassified traces could be a valuable area of future inquiry. Ultimately, the goal is to develop AI tools that can augment clinical decision-making in fetal monitoring, improving diagnostic consistency, reducing clinician workload, and ultimately contributing to better perinatal outcomes. This will likely require interdisciplinary collaboration between AI researchers, obstetricians, and experts in medical imaging.

### Strengths and limitations

This study’s primary strength lies in its direct, comparative evaluation of leading LLMs using real-world, clinician-labeled CTG data. Nevertheless, limitations include the relatively small sample size of sixty CTGs, which may limit the generalizability of the findings. Additionally, the retrospective nature of the study and the use of a single-center dataset may introduce potential biases. Furthermore, while LLMs can process complex input and simulate clinical reasoning, they may still reflect biases from their training data, lack awareness of context-specific nuances, and generate plausible but inaccurate outputs. A significant limitation is the lack of clinical context provided to the LLMs, which is routinely available to human clinicians during CTG interpretation. Real-time applicability, data privacy, and alignment with clinical standards remain ongoing challenges.

## Conclusions

While the potential of AI to assist in medical diagnostics remains promising, our findings reveal significant shortcomings in the ability of general-purpose LLMs to accurately classify CTG traces. Therefore, the integration of LLMs into routine CTG analysis is not yet supported by our findings, emphasizing the continued importance of expert clinical evaluation and the need for targeted research to develop more sophisticated AI tools specifically designed for this critical area of fetal monitoring.

## Supplementary Information

Below is the link to the electronic supplementary material.

Supplementary file1 (DOCX 1490 KB)

Supplementary file2 (DOCX 1977 KB)

## Data Availability

No datasets were generated or analysed during the current study.
