# Supplementary figures and images for: A comparative evaluation of publicly available large language models in the assessment of CTG traces according to the FIGO criteria
Source: Arch Gynecol Obstet. 2025 Aug 21;312(5):1571–80. doi: 10.1007/s00404-025-08145-w (PMC12589333; doi:10.1007/s00404-025-08145-w)

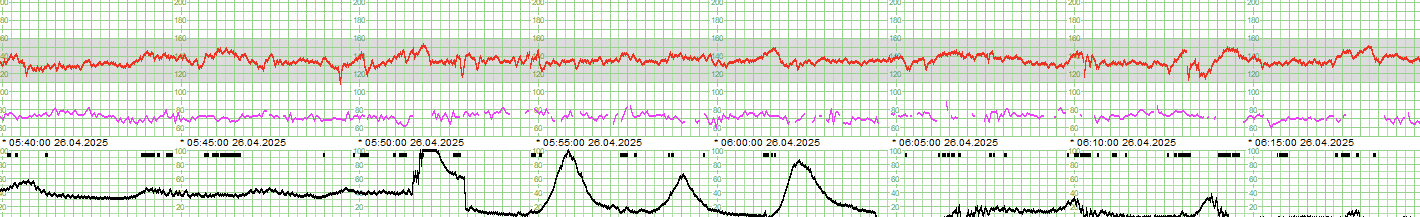


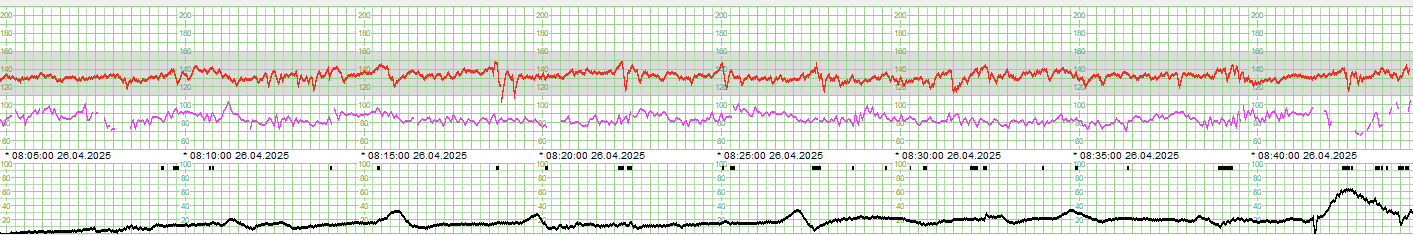


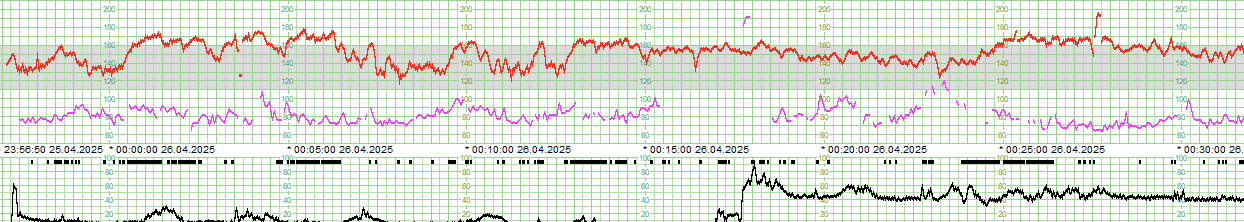


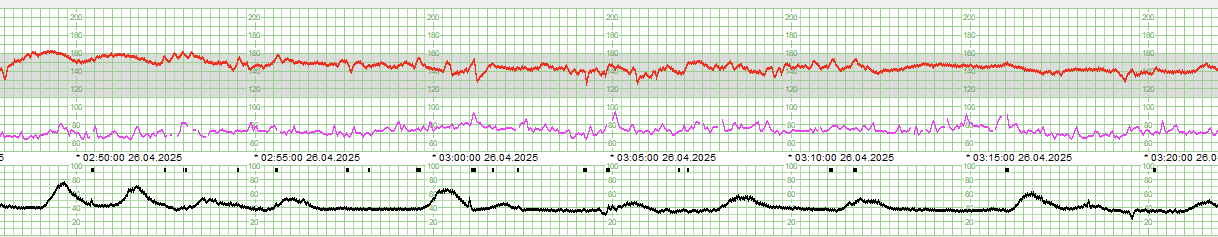


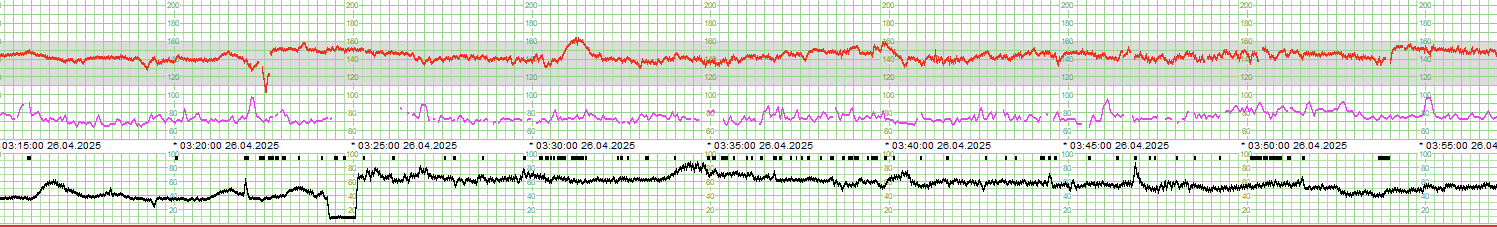


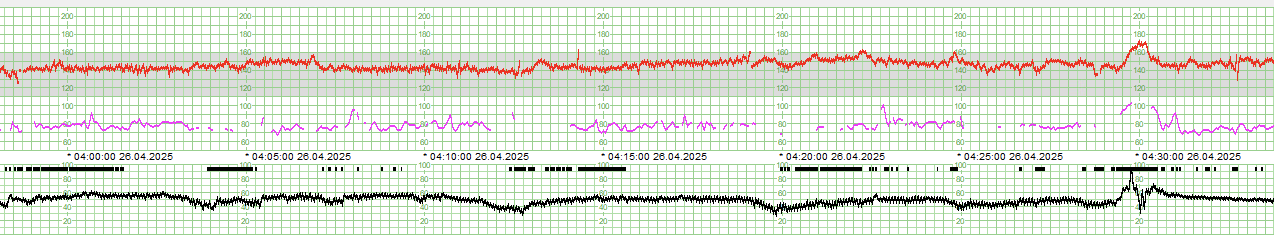


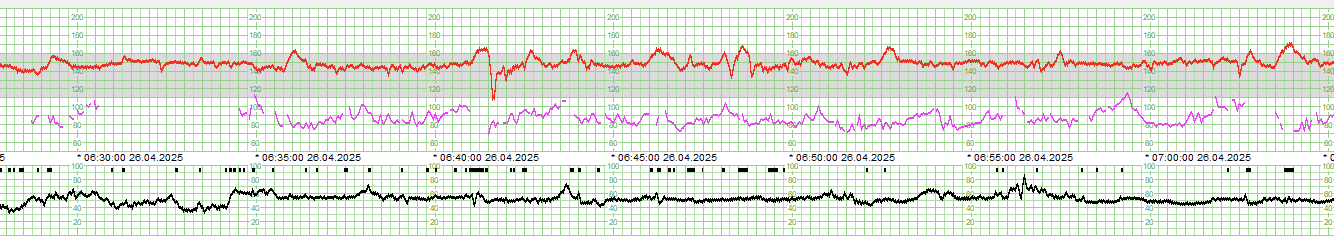


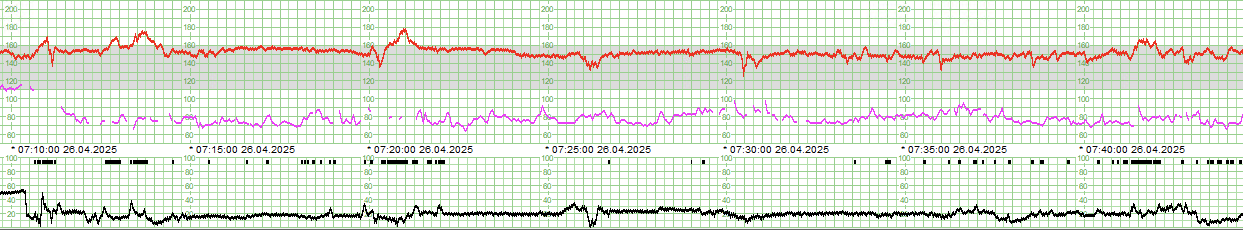


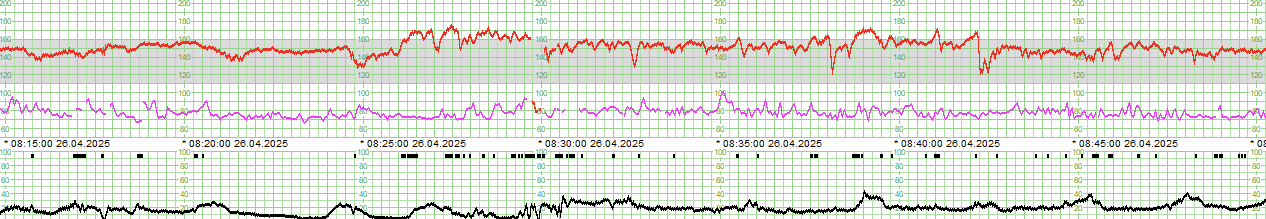


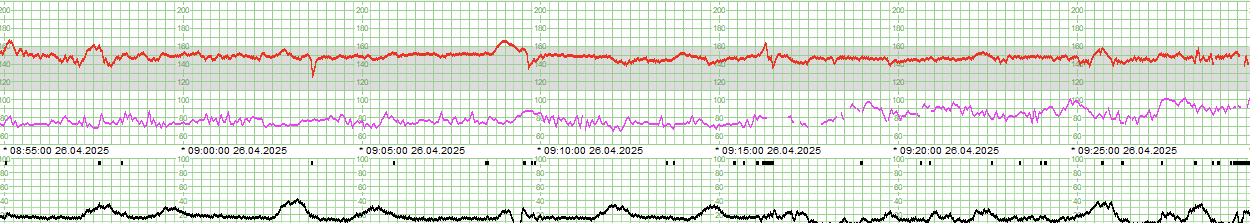


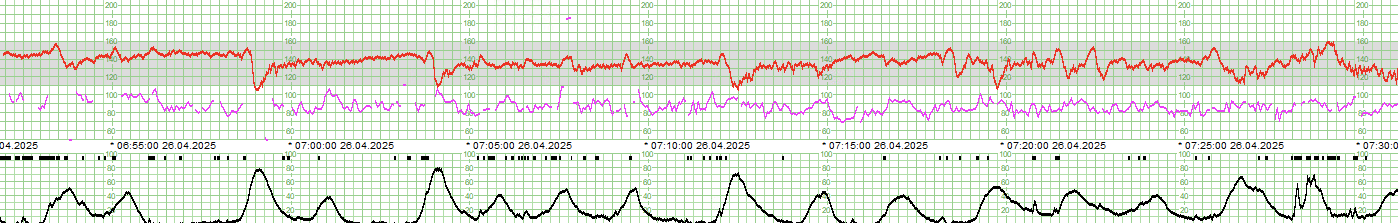


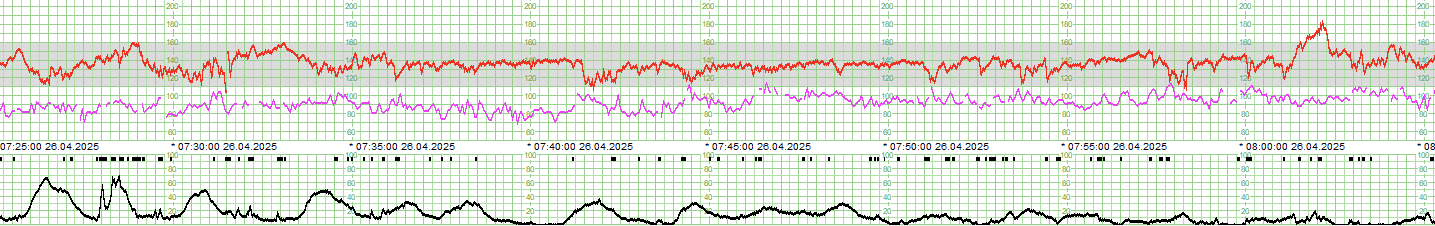


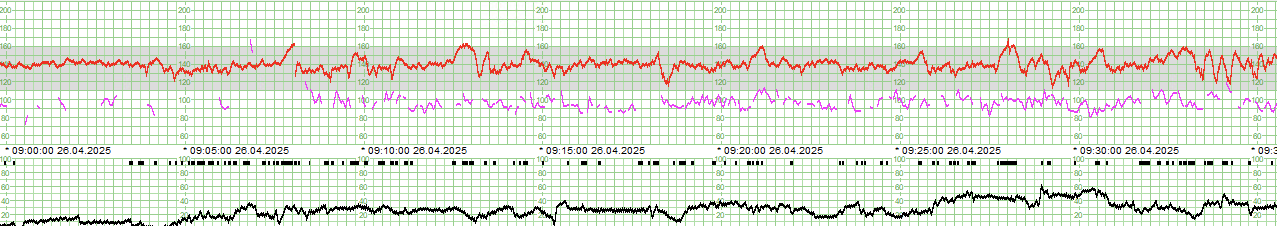


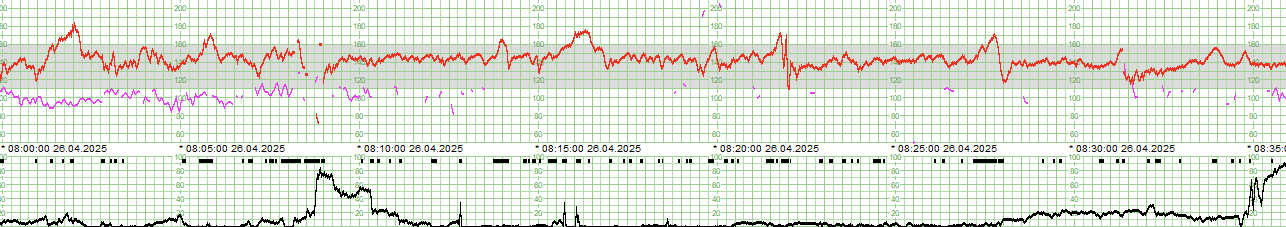


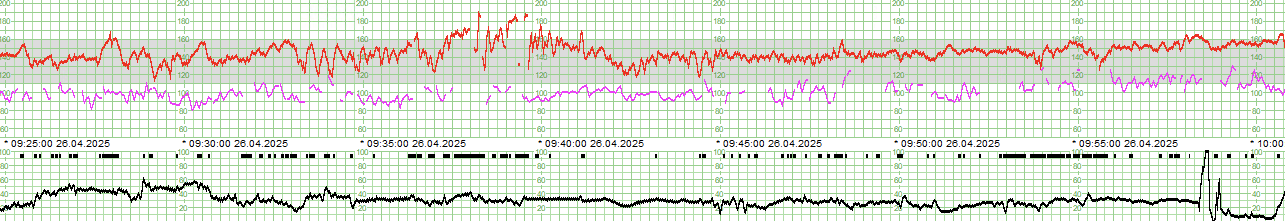


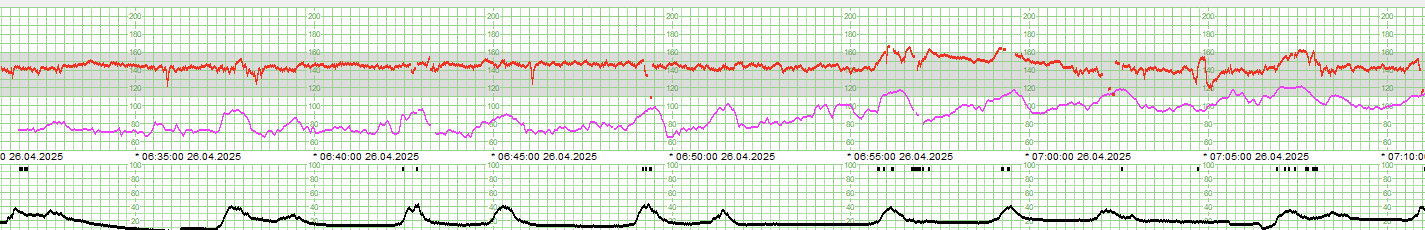


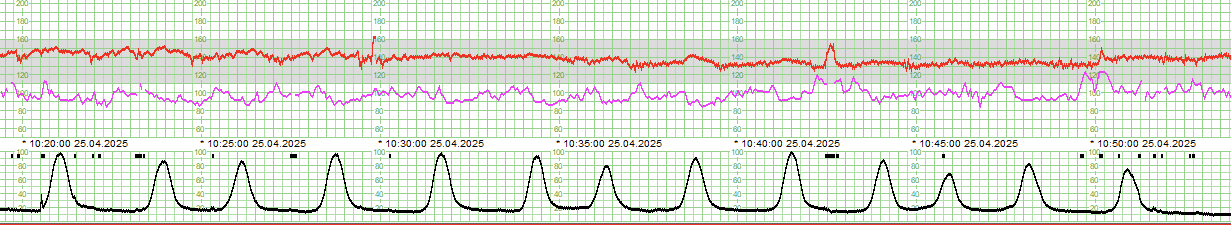


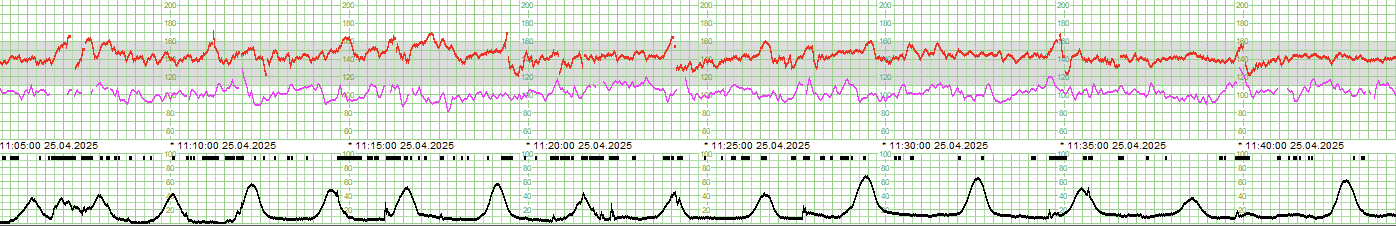


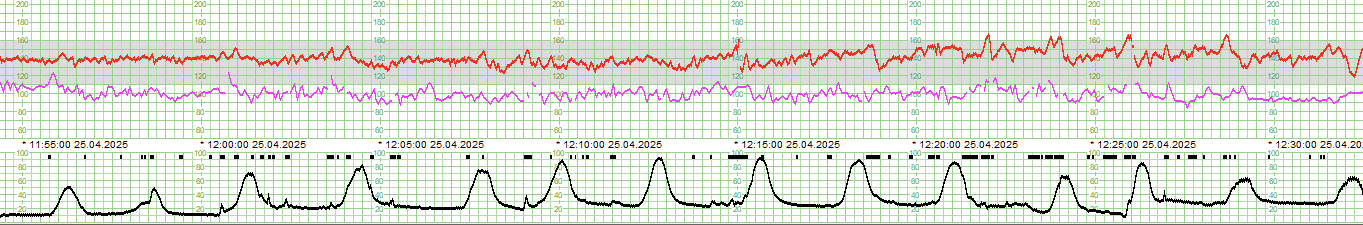


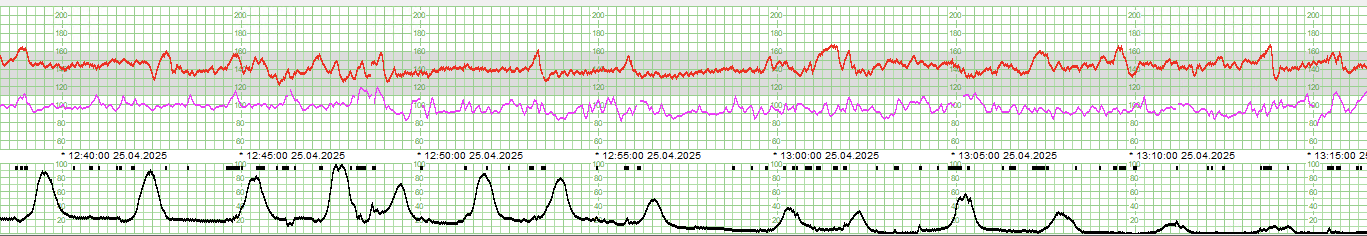


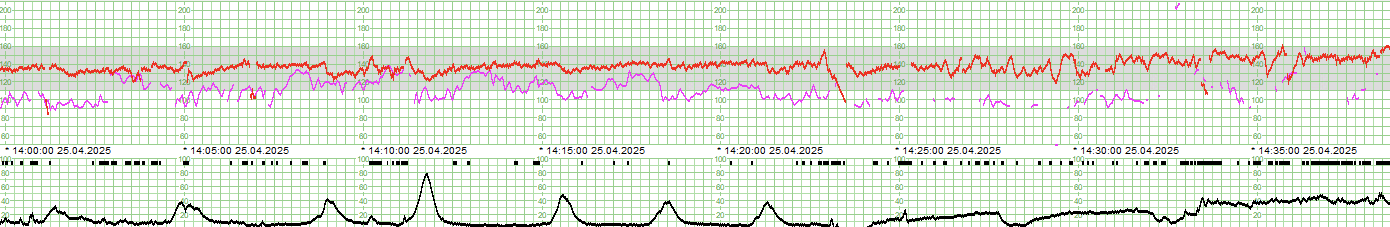


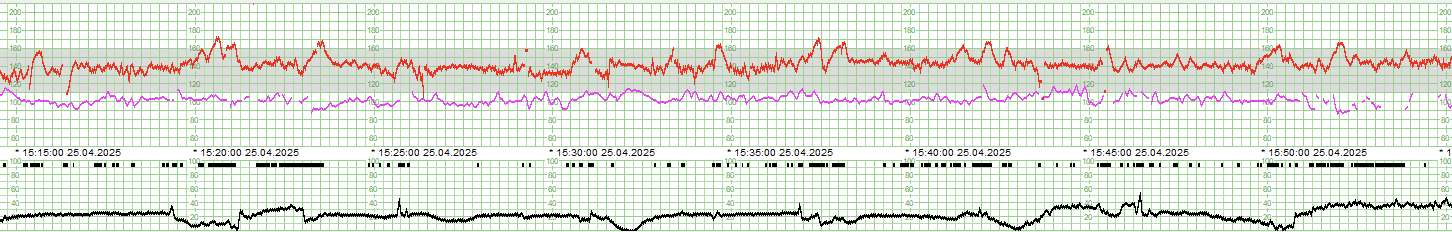


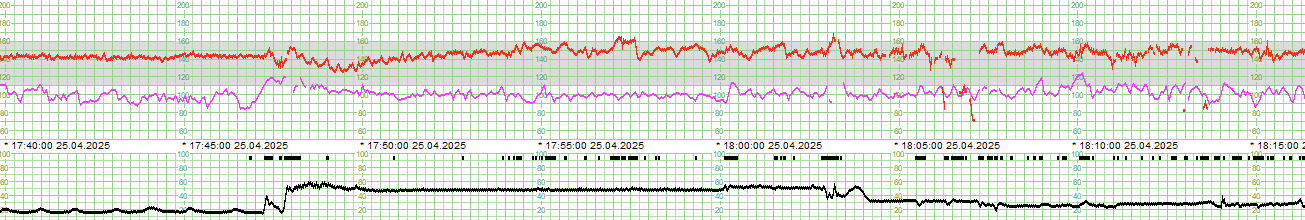


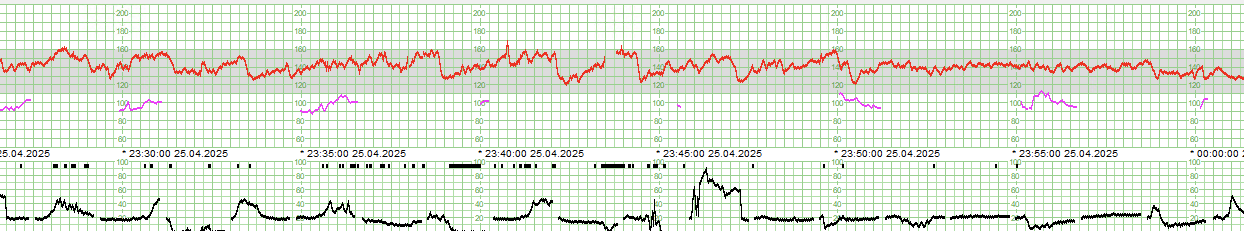


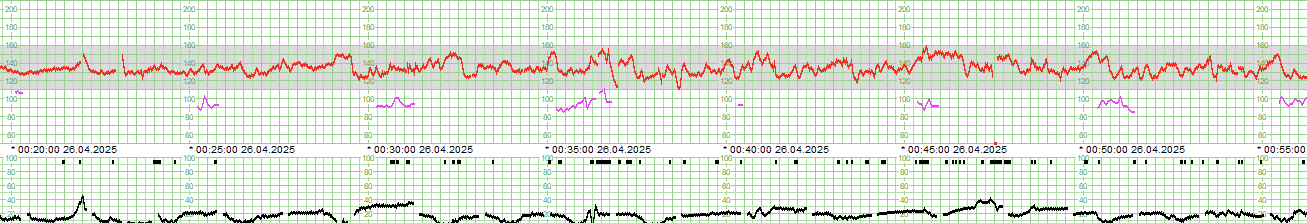


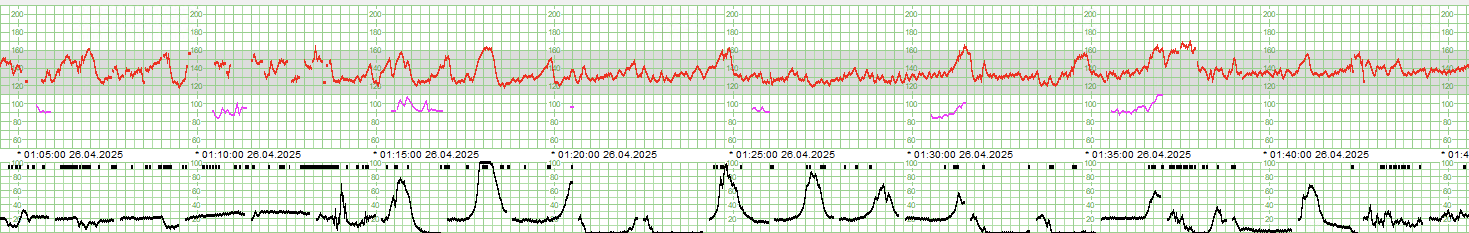


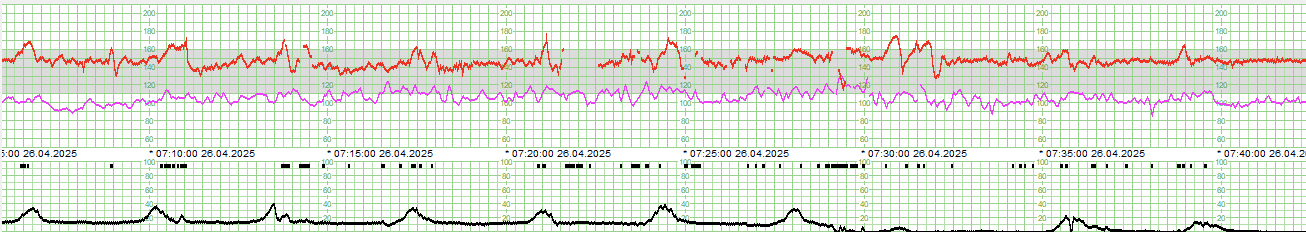


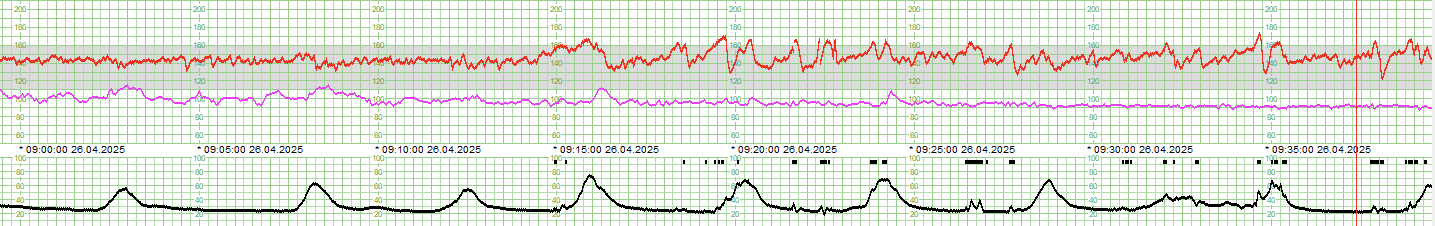


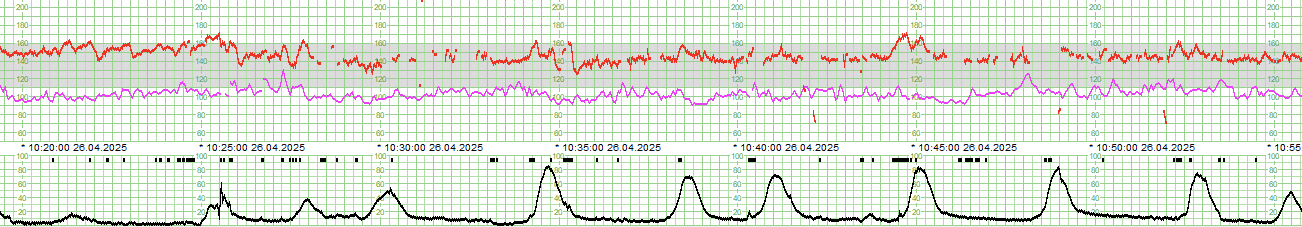


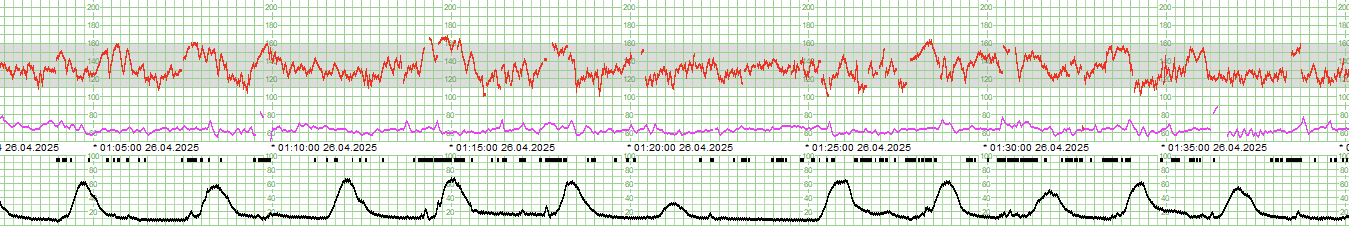

Supplement: Supplementary file 1 — Supplementary file1 (DOCX 1490 KB) [file 404_2025_8145_MOESM1_ESM.docx]

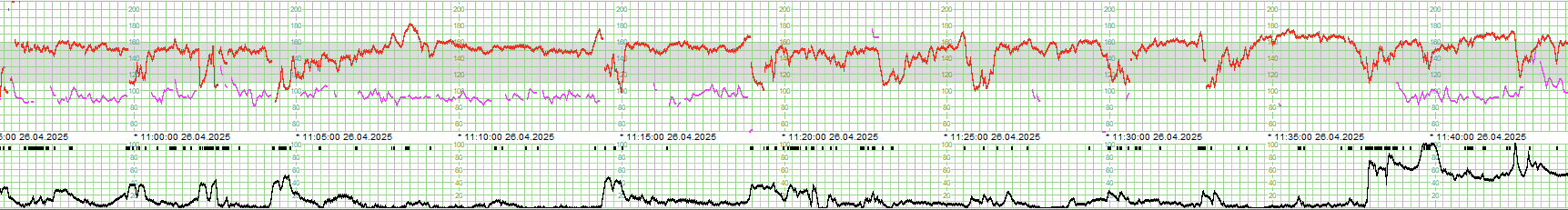


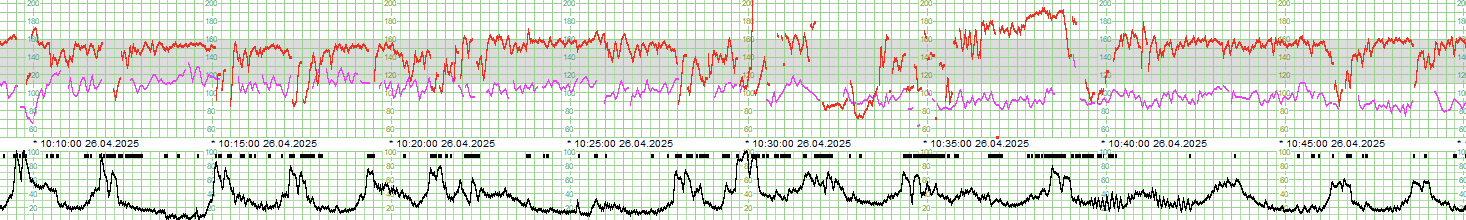


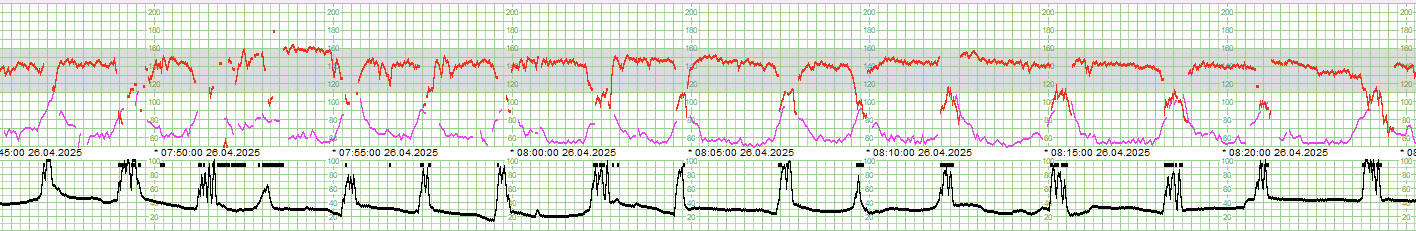


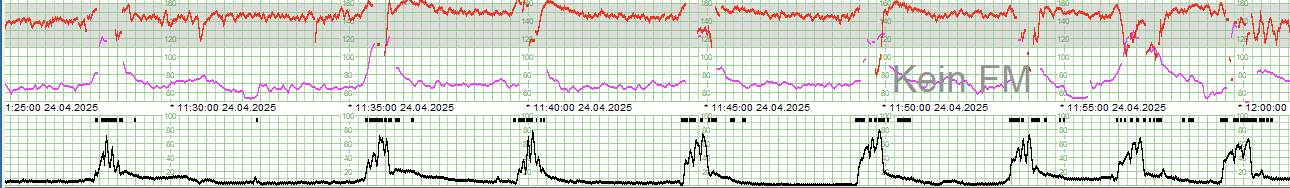


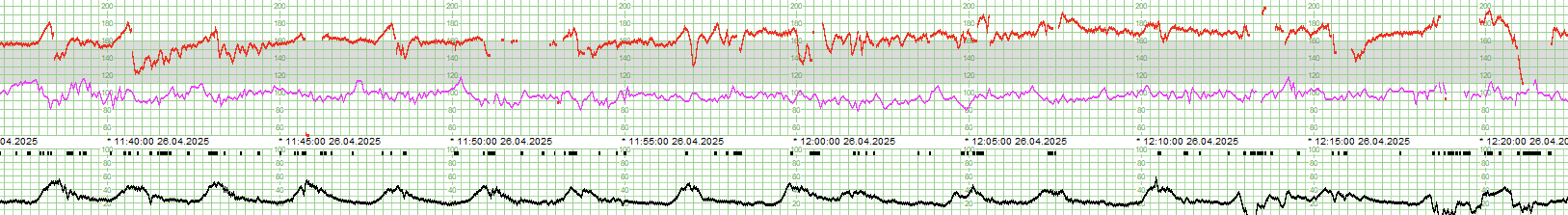


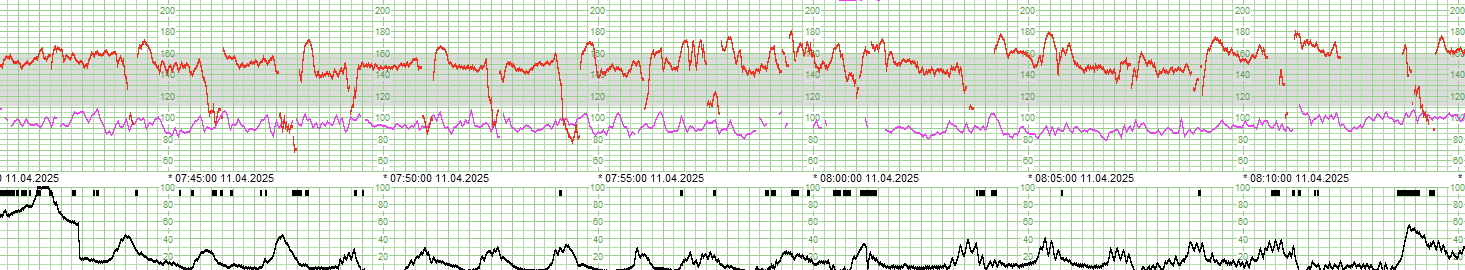


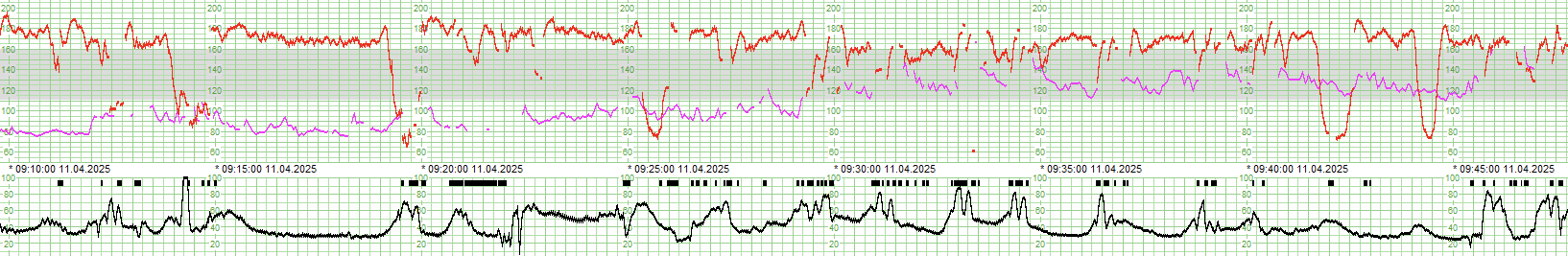


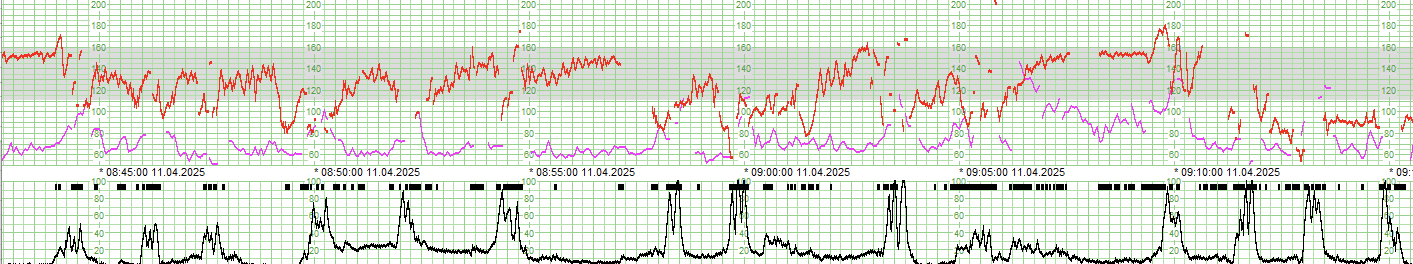


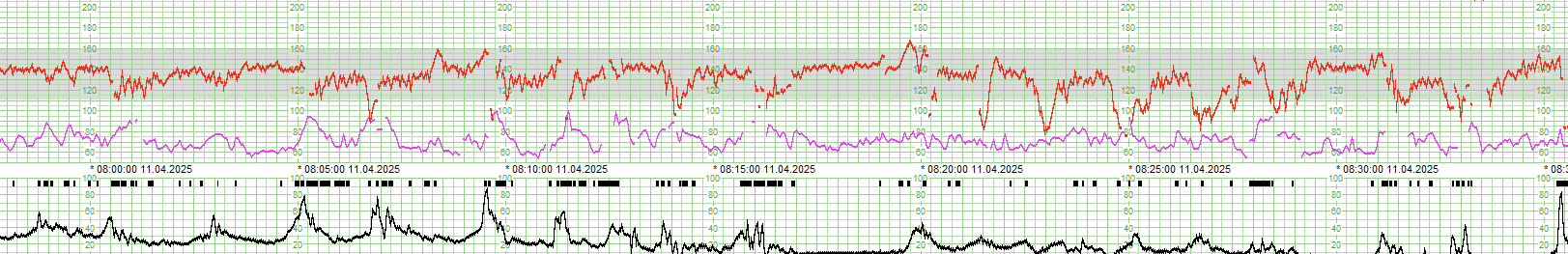


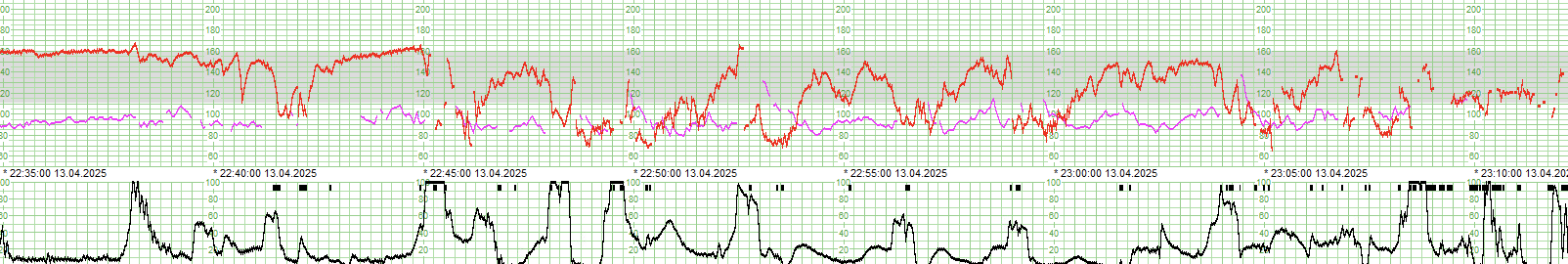


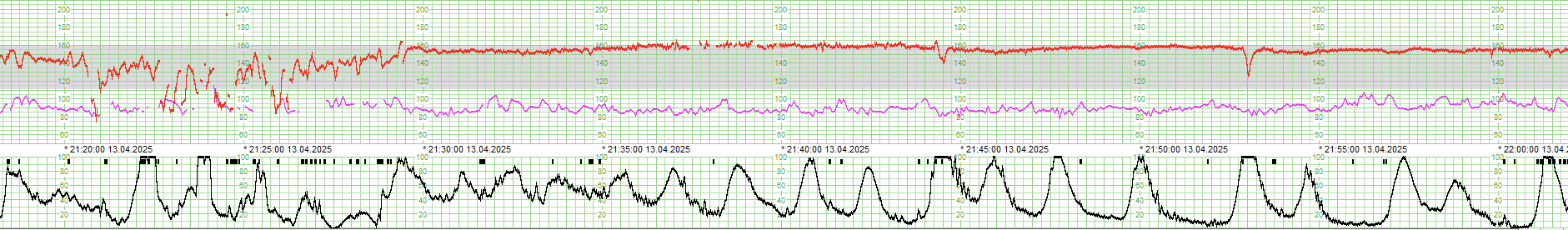


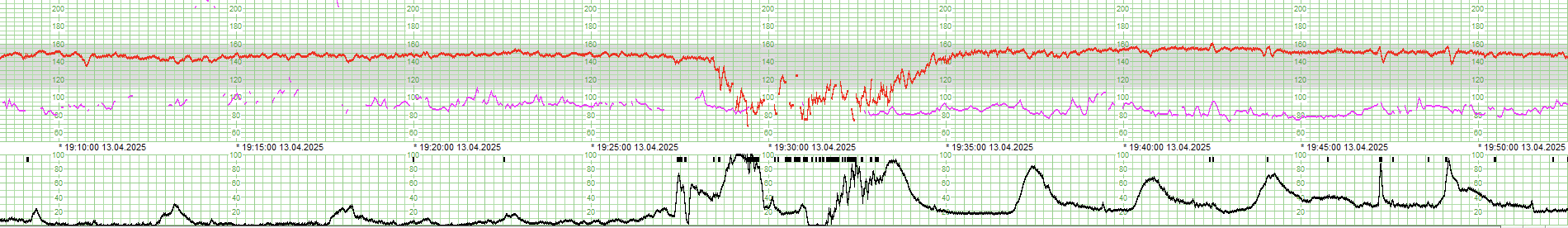


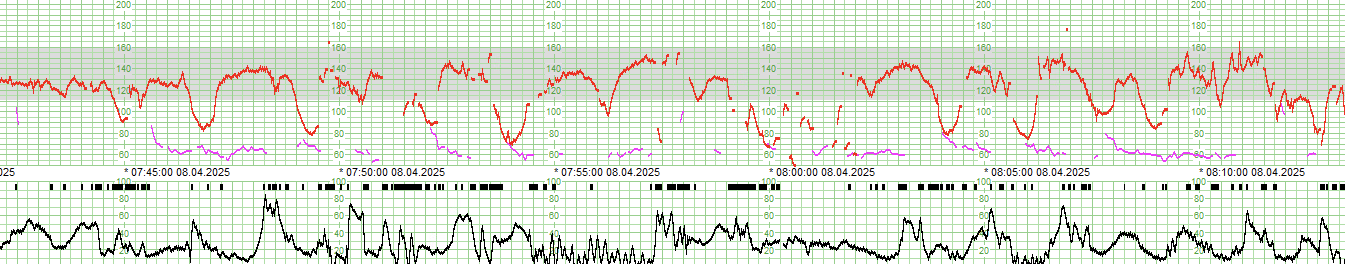


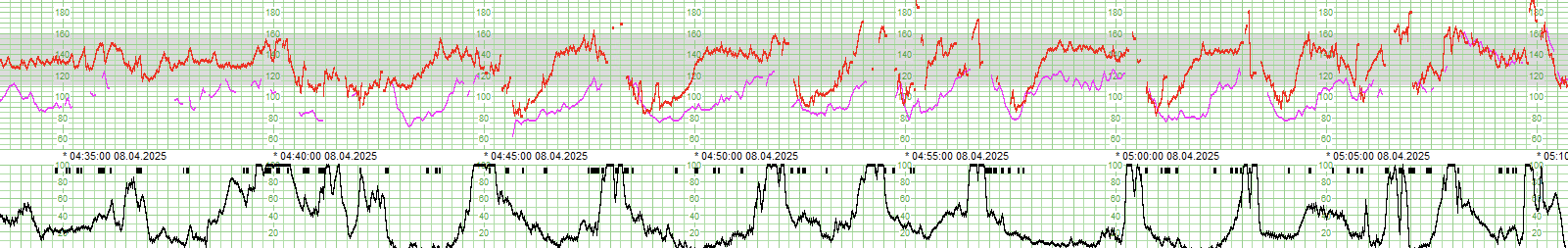


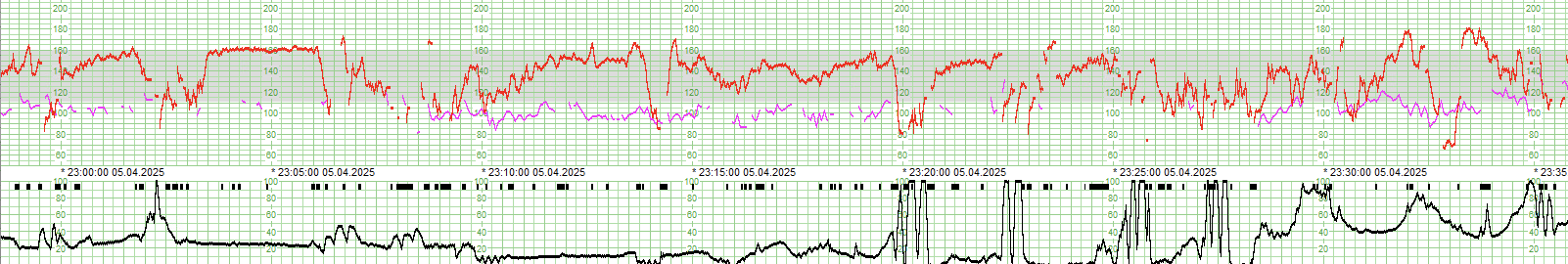


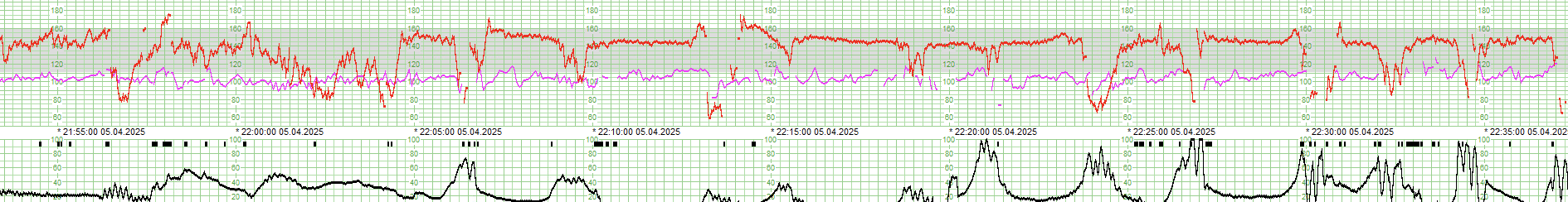


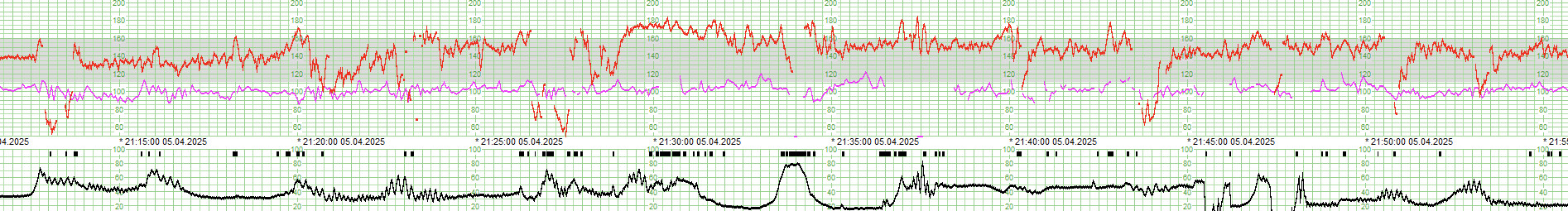


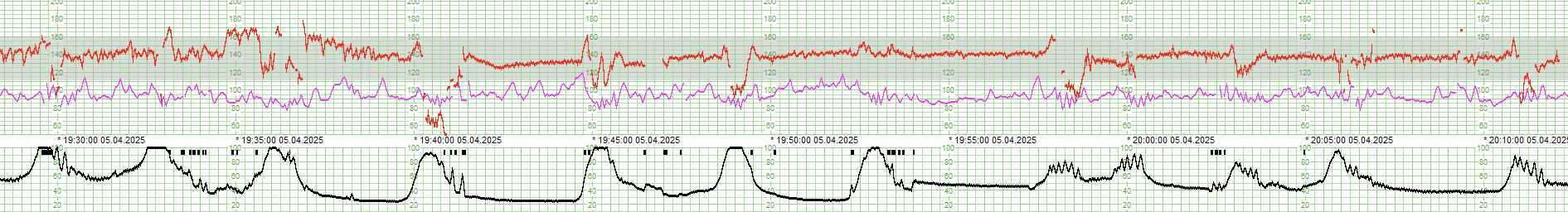


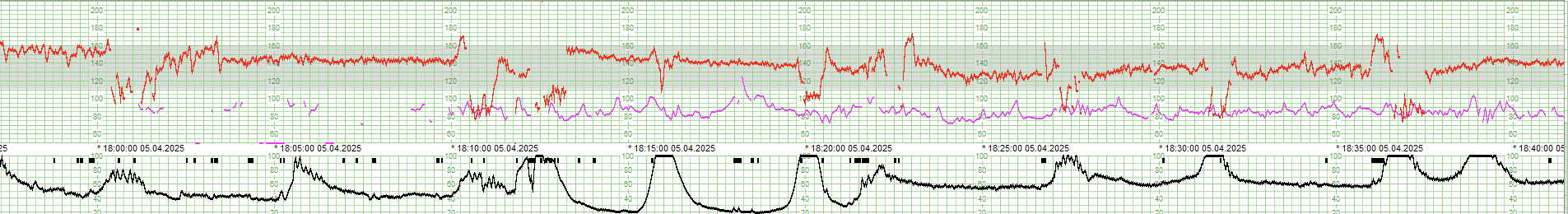


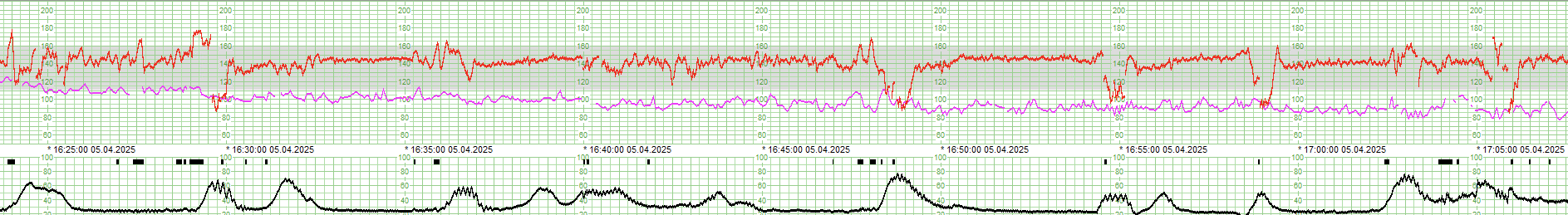


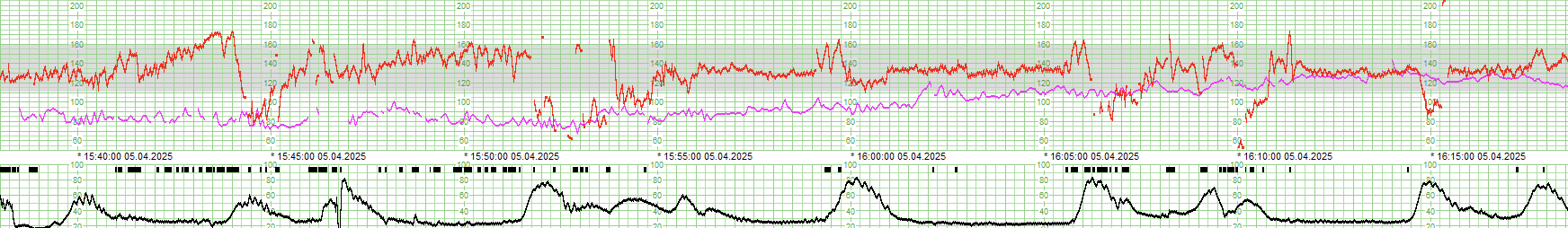


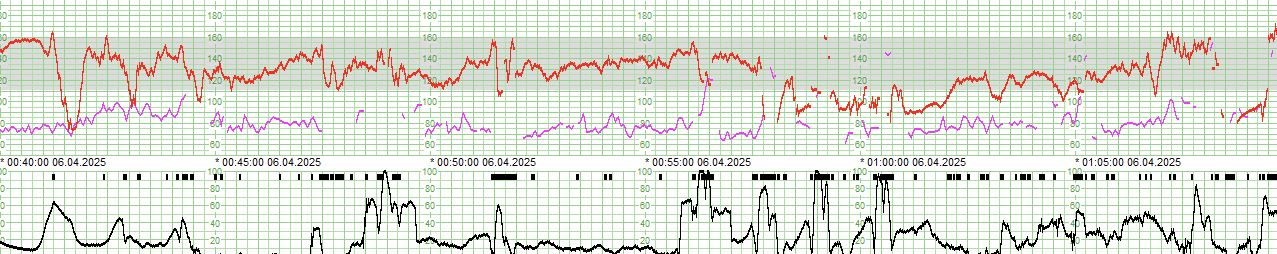


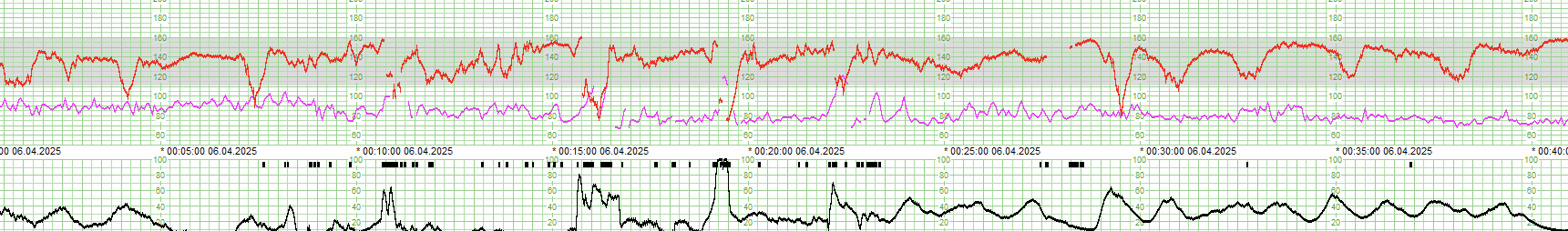


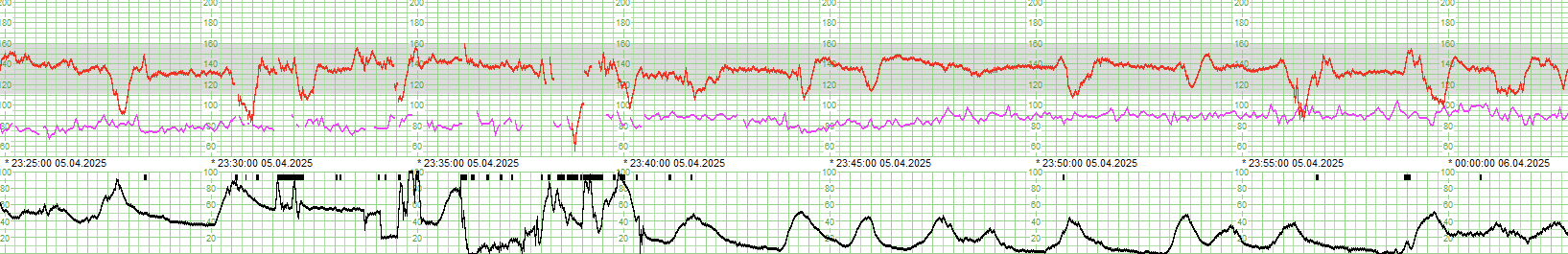


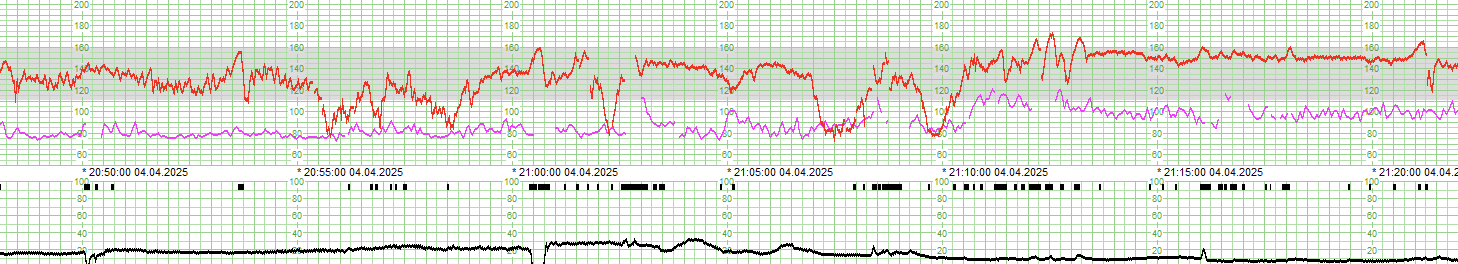


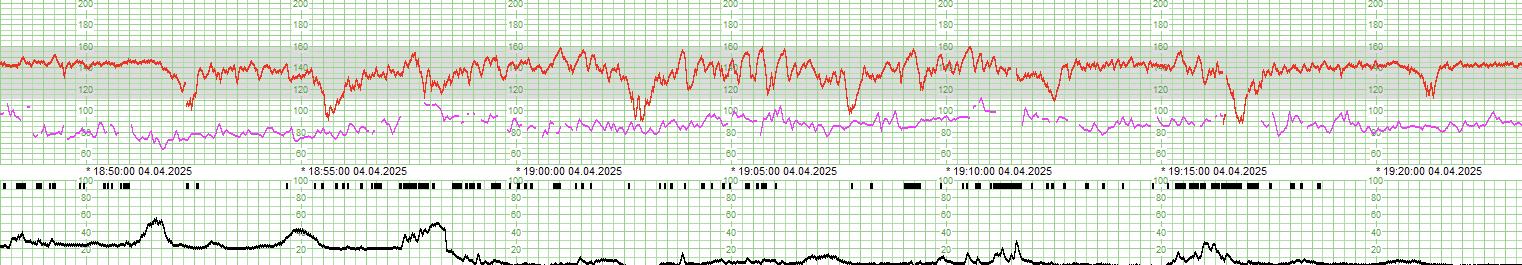


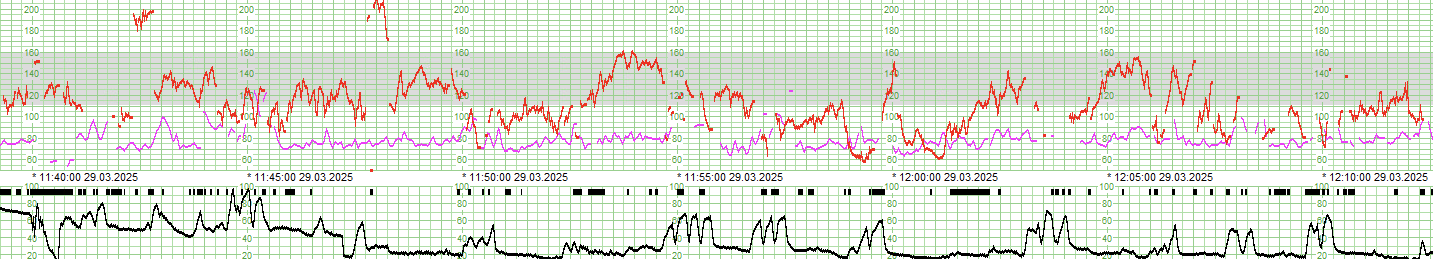


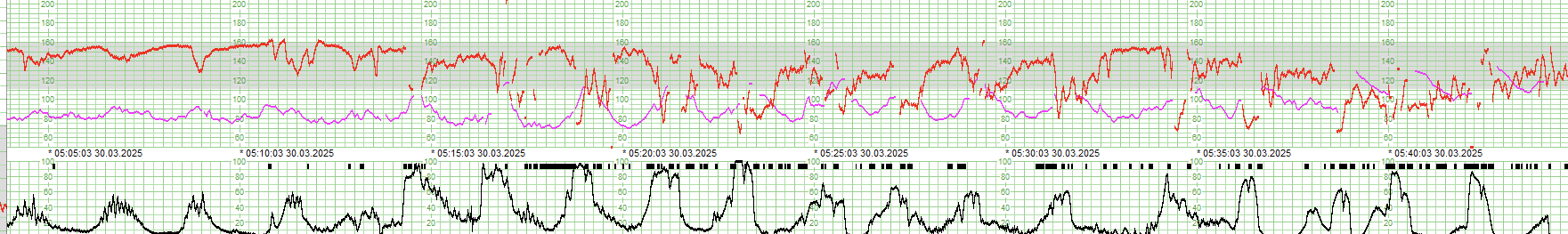


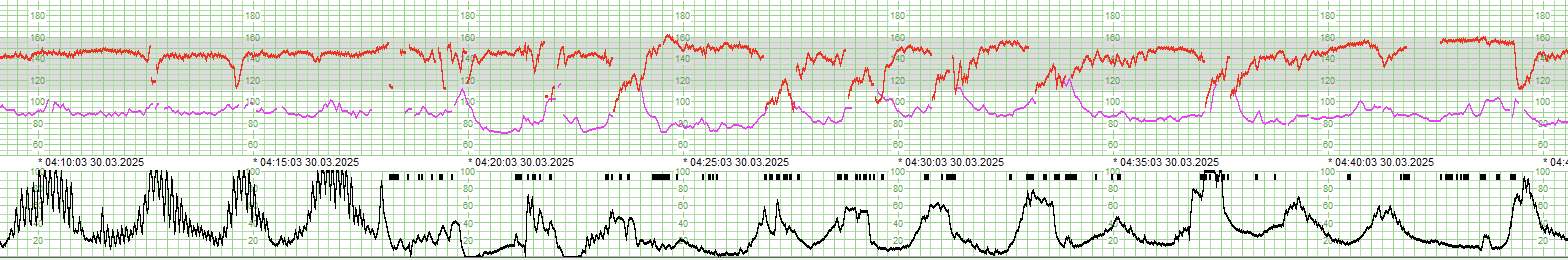


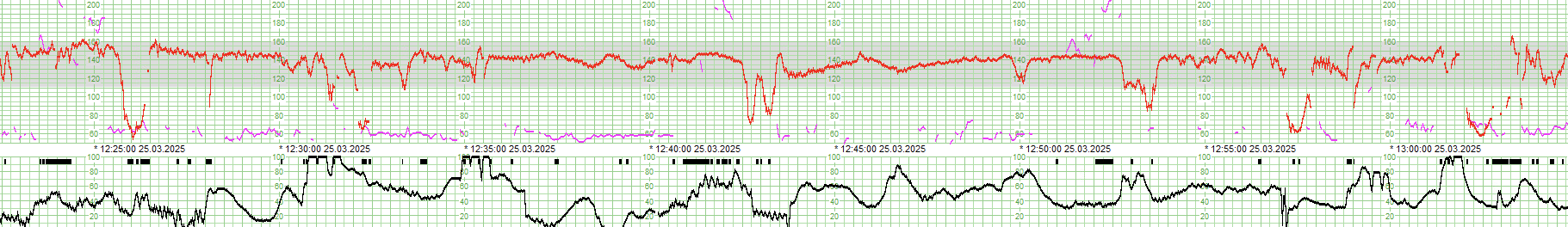

Supplement: Supplementary file 2 — Supplementary file2 (DOCX 1977 KB) [file 404_2025_8145_MOESM2_ESM.docx]
